# Modes-of-Action Related to Repeated Dose Toxicity: Tissue-Specific Biological Roles of PPAR**γ** Ligand-Dependent Dysregulation in Nonalcoholic Fatty Liver Disease

**DOI:** 10.1155/2014/432647

**Published:** 2014-03-18

**Authors:** Merilin Al Sharif, Petko Alov, Vessela Vitcheva, Ilza Pajeva, Ivanka Tsakovska

**Affiliations:** Institute of Biophysics and Biomedical Engineering, Bulgarian Academy of Sciences, Acad. G. Bonchev Street, Building 105, 1113 Sofia, Bulgaria

## Abstract

Comprehensive understanding of the precise mode of action/adverse outcome pathway (MoA/AOP) of chemicals becomes a key step towards superseding the current repeated dose toxicity testing methodology with new generation predictive toxicology tools. The description and characterization of the toxicological MoA leading to non-alcoholic fatty liver disease (NAFLD) are of specific interest, due to its increasing incidence in the modern society. Growing evidence stresses on the PPAR**γ** ligand-dependent dysregulation as a key molecular initiating event (MIE) for this adverse effect. The aim of this work was to analyze and systematize the numerous scientific data about the steatogenic role of PPAR**γ**. Over 300 papers were ranked according to preliminary defined criteria and used as reliable and significant sources of data about the PPAR**γ**-dependent prosteatotic MoA. A detailed analysis was performed regarding proteins which PPAR**γ**-mediated expression changes had been confirmed to be prosteatotic by most experimental evidence. Two probable toxicological MoAs from PPAR**γ** ligand binding to NAFLD were described according to the Organisation for Economic Cooperation and Development (OECD) concepts: (i) PPAR**γ** activation in hepatocytes and (ii) PPAR**γ** inhibition in adipocytes. The possible events at different levels of biological organization starting from the MIE to the organ response and the connections between them were described in details.

## 1. Introduction

Multiple or repeated administration of many chemicals may not produce immediate toxic effects but due to their accumulation in tissues or other mechanisms of homeostasis perturbation, results in delayed effects [1]. Repeated dose toxicity comprises these adverse general toxicological effects which occur as a result of repeated daily dosing with or exposure to a substance for a specified period up to the expected lifespan of the test species [2]. The traditional* in vivo* repeated dose toxicity tests, although still widely used, have a number of limitations [3]. The modern toxicology concepts are based on comprehensive knowledge about biological pathways and their relationship to adverse effects at the organ and higher levels. These concepts allow building alternative models (*in vitro* and computational) to describe the adverse effects [4]. They are at the heart of initiatives such as SEURAT-1 (http://www.seurat-1.eu) and TOX21 (http://www.epa.gov/ncct/Tox21/) and are based on the methodology of the Adverse Outcome Pathway (AOP). The AOP regulatory assessment framework has been provided to collect, organize, and evaluate relevant information about chemical, biological, and toxicological effect of chemicals [5]. It supports the use of a mode-of-action (MoA) basis involving description and characterization of the key cytological and biochemical events that are both measurable and necessary to the observed effect.

**Figure 1:**
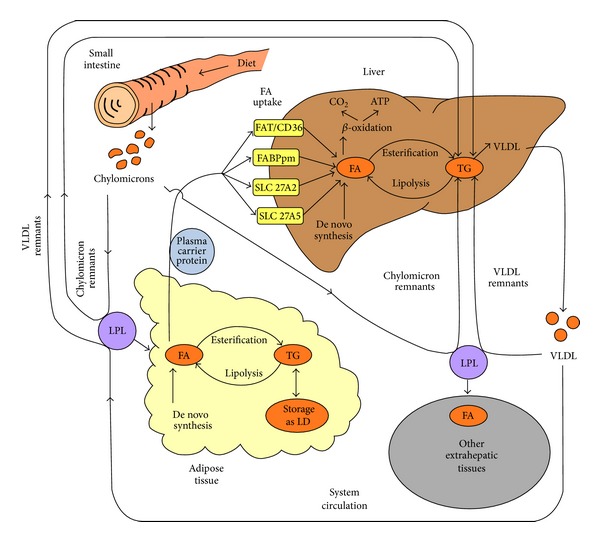
A simplified presentation of the fatty acids transport, metabolism, and fate in the human organism. FAT/CD36: fatty acid translocase/cluster determinant 36; FABPpm: plasma membrane fatty acid binding protein; SLC 27A2: solute carrier family 27 (fatty acid transporter), member 2; SLC 27A5: solute carrier family 27 (fatty acid transporter), member 5; FA: fatty acids; TG: triglycerides; VLDL: very low-density lipoprotein; LPL: lipoprotein lipase; LD: lipid droplet.

Liver is one of the organs that are highly exposed to many potentially toxic substances and therefore a frequent target for toxicity. It has a central role in the lipid homeostasis and its primary function is fat redistribution instead of storage, the latter being typical for adipose tissue (Figure 1). The liver damage can be a result of direct hepatocyte damage, hepatic tumor, and/or accumulation of lipids or phospholipids (fatty liver disorder). The nonalcoholic fatty liver disease (NAFLD) is a medical condition characterized by significant lipid deposition in the hepatocytes [6]. NAFLD is a common cause of chronic liver injury; thus, in view of repeated-dose hepatotoxicity, its pathogenesis is of particular interest, with emphasis on the mode and site of action of potential chemical inducers. Different molecular initiating events (MIEs) influence the onset and progression of these toxic effects [7].

Peroxisome proliferator-activated receptor gamma (PPAR*γ*) has been recently proposed as one of the receptors involved in the MIE for liver steatosis (the early manifestation of NAFLD) [7]. PPAR*γ* is responsible for the regulation of adipogenesis (adipocyte proliferation and differentiation), lipid and glucose homeostasis, inflammatory responses, vascular functions, and placental development [8–10]. The modulation of PPAR*γ* function by ligand binding reflects on its genomic activity (transactivation and transrepression). The up-regulated genes are associated with lipid transport, metabolism, storage, and adipogenesis [11, 12]. The down-regulated genes typically include those involved in adaptive inflammatory responses. Several transrepression mechanisms have been reported [12].

PPAR*γ* has two isoforms, PPAR*γ*1 and PPAR*γ*2, differing by a thirty amino acid N-terminal extension present in PPAR*γ*2. While PPAR*γ*1 is expressed in multiple tissues including liver, adipocytes are the most likely site of PPAR*γ*2 expression [13, 14].

In this study, we present a comprehensive analysis of the data reported in the scientific literature about the role of PPAR*γ* in the pathogenesis of NAFLD. Based on these data and according to the OECD guidelines [5], the main possible MoAs starting from xenobiotic interaction with PPAR*γ* as a MIE, passing through downstream transcriptional dysregulation, and resulting in the first two stages of NAFLD, namely, liver steatosis and nonalcoholic steatohepatitis (NASH), are outlined.

## 2. Methodology of the Analysis

In order to clarify the role of PPAR*γ* ligand binding in the MoA of NAFLD, we have summarized and analyzed the experimental data from studies on hepatocytes, as well as adipocytes.

Over 300 papers retrieved from NIH PubMed system (http://www.ncbi.nlm.nih.gov/pubmed) were screened and ranked according to the following general criteria:

- completeness of the model description: type of experiment (*in vivo *or* in vitro*), species or cell line used, and genetic properties of the studied subjects which could support a causal link between the MIE and the adverse outcome;
- relevance of the presented experimental evidence to studied MoA: availability of results from biochemical, histological, or other assays that are qualitatively or quantitatively associated with commonly accepted markers of NAFLD;
- availability of sufficient data for categorization of the experimental observations as key molecular events (initiating or intermediate) of studied MoA: experimentally-induced (by diet, pharmacological treatment, or genetic techniques) changes in PPAR*γ* activity and/or expression accompanied by changes in the expression of PPAR*γ* target proteins.

**Figure 2:**
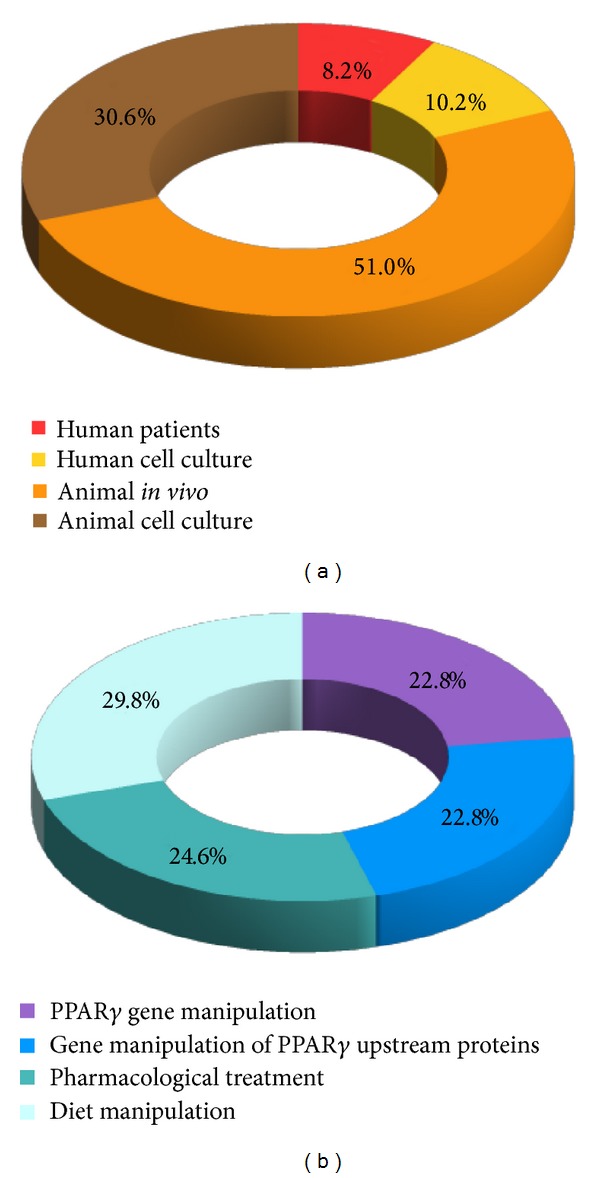
Major categories of subjects (a) and experimental approaches (b) in the selected papers.

The analysis of the collected papers was done in several steps. First, the initial pool was filtered for availability of information about potential toxicity pathways and target proteins related to them and all papers containing such data were scored. Next, scores were given depending on the investigated subjects (humans or animals) with a higher score for papers reporting human data. The papers that met at least one of the following criteria were selected for further evaluation: evidence for PPAR*γ* dysregulation, relation to the selected endpoints (steatosis or steatohepatitis), or intermediate events preceding these endpoints. The selected papers constituted the core set, which was further extended by additional more specific literature search on PPAR*γ*, the target protein, and the toxicity pathway. The final set contained 72 papers, among them 26 reviews. Supplementary Table 1 in Supplementary Material (available online at http://dx.doi.org/10.1155/2014/432647) classifies the available data in all 72 papers in relation to: the studied subjects (human patients, human cell cultures, animals* in vivo*, and animal cell cultures), the experimental approaches (PPAR*γ* overexpression, PPAR*γ* overexpression and pharmacological treatment; PPAR*γ* knockout/knockdown; PPAR*γ* knockout/knockdown and pharmacological treatment; pharmacological treatment; diet manipulation; gene manipulation of PPAR*γ* upstream proteins; gene manipulation of PPAR*γ* upstream proteins and pharmacological treatment). The papers dealing with the AOP methodology, reviews, and research articles containing background information (receptor structure, up- and downstream proteins' functions, etc.) are given in the last two columns.  Figure 2 summarizes the data in Supplementary Table 1.

The analysis of the selected papers served as a basis for building the blocks in the proposed MoA.  Table 1 exemplifies a summary of the main findings in these papers related to one of the most studied PPAR*γ* target proteins CD36.

## 3. Results and Discussion

The coordinated cellular regulation of the lipid metabolism pathways and the dynamic balance of the intertissue lipid exchange are crucial for the whole-body lipid homeostasis. NAFLD stems from abnormalities such as increased fatty acid (FA) uptake; increased* de novo* FA synthesis; decreased FA oxidation; or impaired VLDL secretion [15, 25, 26].

The effects of PPAR*γ* dysregulation on the liver remain under debate, with some studies showing that it promotes hepatic steatosis through up-regulation of genes involved in lipid uptake and storage and others showing that it prevents hepatic steatosis and fibrosis, possibly by sequestering FAs in adipose tissue and preventing hepatic stellate cell activation [13, 14]. However, growing evidence stresses on the importance of PPAR*γ* in pathogenesis of NAFLD [16, 25, 27–30].* In vitro* and* in vivo* experiments on animal models have confirmed that hepatic overexpression and/or activation of the receptor by an agonist triggers undesirable up-regulation of various lipogenic target genes [16–18]. Recently, it has been demonstrated that liver-specific knockout of PPAR*γ* could prevent fatty liver down-regulating genes coding for lipogenic and fatty acid transport proteins [17]. PPAR*γ* knockdown by interfering RNA also reduces the liver concentration of triglycerides (TG) [19]. Detrimental hepatic PPAR*γ* expression as a consequence of genetic alterations has been reported, where receptor activation was shown to initiate massive liver steatosis and hepatocyte proliferation [31]. PPAR*γ* gene nucleotide variations have also been reported to affect hepatic steatosis, often in relation to partial lipodystrophy [11, 32].

In the ideal scenario, the MIE as a primary anchor or “the foundation” of the AOP should be well-defined. However, not only the potential of a chemical to elicit that event should be recognized but also the likely site of action (in terms of the receptor tissue localization) should be noted [5]. In this particular case, PPAR*γ* dysregulation by ligand-dependent activation or inhibition may result in the same adverse outcome but the site of action could be different (hepatocyte versus adipocyte).

### 3.1. PPAR*γ* Ligand-Dependent Activation in Hepatocytes

The relevance of the PPAR*γ* ligand-dependent activation as a MIE in NAFLD-related toxicity pathways has been supported by data about prosteatogenic effects of PPAR*γ* agonists (synthetic: rosiglitazone and pioglitazone; endogenous: palmitate and oleate) and/or overexpression of PPAR*γ* in the liver [16, 17, 27, 30] as well as by the observed protective effect against hepatic steatosis of PPAR*γ* antagonists (BADGE, GW9662) or hepatocyte-specific PPAR*γ* knockout or knockdown [20, 30].

**Figure 3:**
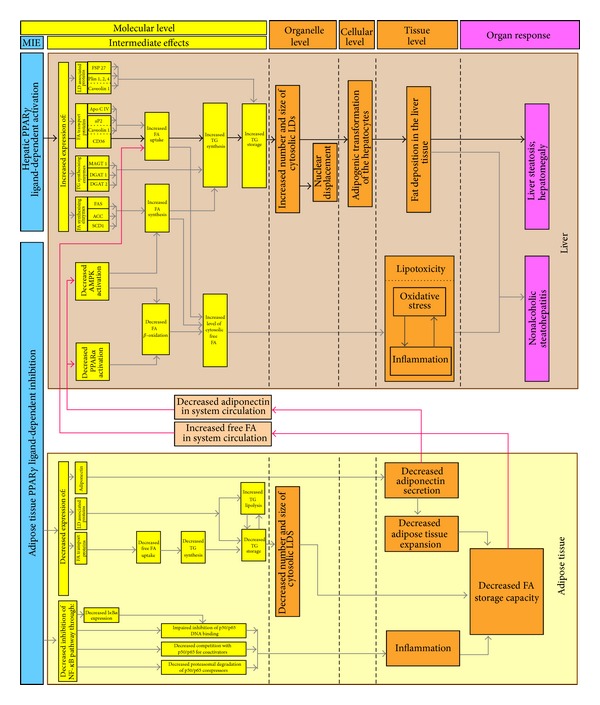
Probable MoAs leading from tissue-specific ligand-dependent PPAR*γ* dysregulation to NAFLD. FSP27/CIDE-C: fat-specific protein 27/cell death-inducing DFF45-like effector; Plin 1, 2, 4: Perilipins 1, 2, and 4; ApoCIV: apolipoprotein C IV; aP2: adipose fatty acid binding protein; FAT/CD36 (or just CD36): fatty acid translocase/cluster determinant 36; FAS: fatty acid synthase; ACC: acetyl-CoA carboxylase; SCD1: stearoyl-CoA desaturase1; MGAT1: monoacylglycerol O-acyltransferase 1; DGAT1: diglyceride acyltransferase 1; DGAT2: diglyceride acyltransferase 2.

On the basis of the literature data reviewed, four main toxicity pathways from hepatic PPAR*γ* ligand-dependent activation to NAFLD were outlined for inclusion in MoA: uptake of FA*, de novo* synthesis of FA, TG synthesis, and lipid storage. The results of the literature analysis are summarized in Figure 3.

The lipogenic PPAR*γ* target proteins include enzymes involved in different rate limiting stages of the synthesis of FAs (FAS, ACC, SCD1) and TGs (MGAT1, DGAT1, DGAT2) [16, 17, 21, 22, 24]. Among the lipid droplet-associated proteins (LD proteins) considered to be prosteatotic are FSP27/CIDE-C, Plin 1, 2, 4, Caveolin 1 [21, 25, 29, 33–35]. The group of lipid transport/binding proteins includes ApoCIV, aP2, Caveolin 1, and FAT/CD36 [15–24, 35–37]. The analysis of the studies regarding the target proteins that could be up-regulated in response to this MIE points to CD36, aP2, and FSP27 as the most completely characterized prosteatotic factors.

The FAT/CD36 (FA translocase/cluster determinant 36) protein is a member of the class B scavenger receptor family. It is known for its role in the uptake of oxidized low-density lipoprotein by macrophages and uptake of FAs by adipose tissues, skeletal muscle, and heart. Equally important function of CD36 in the uptake of FAs in the liver and the pathogenesis of fatty liver disease has recently been outlined [25]. Thus, CD36 and its transcriptional regulators can represent novel therapeutic targets for the prevention and management of fatty liver disease. Additionally, plasma soluble CD36 has recently been proposed as a new biomarker of a phenotype of insulin resistance, carotid atherosclerosis, and fatty liver in a study of healthy nondiabetic subjects [38].

CD36 localizes on the cell surface caveolae, as well as on intracellular vesicles and mitochondria, where it interacts with carnitine palmitoyl transferase 1, the key mitochondrial enzyme regulating FA transport, and oxidation in mitochondria. Mitochondrial CD36 content correlates with mitochondrial FA oxidation in human muscle and is increased by treatment with rosiglitazone [39–41]. To date, several transcriptional regulators of CD36 are reported, including ligand-sensing and lipogenic transcriptional factors, such as cytosolic aryl hydrocarbon receptor (AhR), and several nuclear hormone receptors such as pregnane X receptor (PXR), liver X receptor (LXR), and PPAR*γ* [25]. In particular, adipogenic transformation of liver and exacerbation of steatosis have been strongly associated with the PPAR*γ*-mediated elevation of CD36 mRNA and protein levels [15, 19, 37].

**Figure 4:**
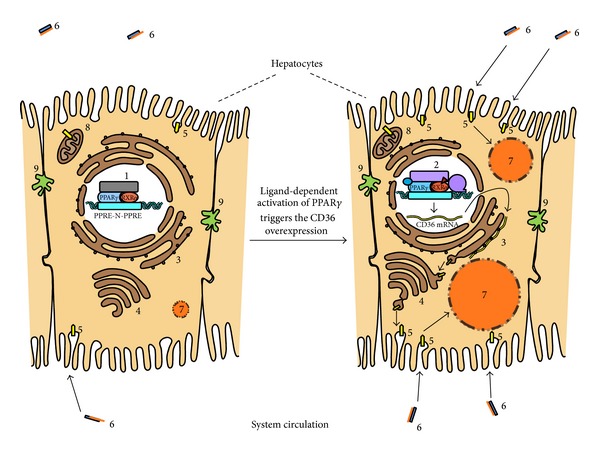
Model of ligand-dependent PPAR*γ* activation as a potential MIE for liver steatosis through CD36 mediated excessive FA uptake and consequent hepatic TG accumulation. (1): PPAR*γ*-RXR*α*-heterodimer interacting with the PPAR*γ* response elements (PPRE-N-PPRE) and transcriptional corepressor complex; (2): ligand-activated PPAR*γ*-RXR*α* heterodimer with transcriptional coactivator complex and RNA pol II; (3): rough endoplasmic reticulum; (4): Golgi complex; (5): FAT/CD36 (fatty acid translocase); (6): plasma fatty acid binding protein (in blue) carrying fatty acid (in orange); (7): growing lipid droplet storing triglycerides and coated with LD associated proteins; (8): mitochondria; (9): bile canaliculus.

A model describing the CD36 mediated toxicity pathway from hepatic PPAR*γ* ligand-dependent activation to increased TG accumulation is presented in Figure 4 as follows: (1) in the absence of ligands (agonists), the heterodimer of PPAR*γ* with retinoid X receptor alpha (RXR*α*) is associated with corepressors turning off gene transcription; (2) the agonist binding induces conformational changes in the receptor followed by replacement of corepressors by coactivators that triggers gene transcription; (3–6) the overexpression and translocation of CD36 to the plasma membrane markedly increase the hepatic uptake of FAs from the circulation; (7) the enhanced esterification of these fatty acids results in increased TG storage in LDs.

An early hypothesis about the mechanism of long-chain FAs transmembrane passage emphasized the interactions of FAT/CD36 and FABPpm (plasma membrane fatty acid binding protein). The latter has been suggested to act as a receptor for long-chain FAs, facilitating the diffusion of the fatty acid-albumin complex through the unstirred fluid layer, while FAT/CD36 was supposed to facilitate FAs flip-flop across the bilayer [42]. The concept about CD36 being a simple transporter was recently questioned as real-time fluorescence measurements revealed a CD36-dependent enhancement of intracellular FA metabolism (e.g., esterification). Thus, a rate increase of FAs uptake mediated by their extensive incorporation into TGs instead of catalyzing the FA translocation across the plasma membrane has been proposed. Although the precise molecular mechanism of the long-chain FAs uptake is still under debate, there is no doubt that CD36 is central to the TG accumulation as HEK293 cells overexpressing CD36 have been shown to accumulate more and larger LDs [43].

Along with the FAs uptake, other key intermediate events are included in the toxicity pathways (Figure 3). They are associated with increased FA synthesis, TG synthesis, and TG storage, all together leading to microvesicular (increased number of LD) or macrovesicular (increased size of LD) steatosis [16, 18–20]. However, LDs are not considered merely as storage depots for superfluous intracellular lipids in times of hyperlipidemic stress, but they are metabolically active organelles involved in cellular homeostasis [44, 45].

Following excessive fat deposition at tissue level, liver steatosis with significant hepatomegaly [20–22] was underlined as one possible organ response, while NASH was incorporated in the MoA as combination of hepatic steatosis and inflammation, the latter stemming from lipotoxicity [20, 23].

### 3.2. PPAR*γ* Ligand-Dependent Inhibition in Adipocytes

PPAR*γ*2 isoform is expressed predominantly in the adipocytes. Its role in fatty acid uptake into adipocytes and adipocyte differentiation has been well defined in experiments with thiazolidinediones and other insulin-sensitizing agents that are potent PPAR*γ* agonists. Activation of PPAR*γ* promotes sequestration of lipids into adipose tissue that has been recognized to affect circulating levels of triglyceride and free FA, with secondary decrease of hepatic lipid uptake and lipotoxicity in the liver [46–48].

Natural occurrence of mutant PPAR*γ* alleles that impair its native function has been considered extremely informative for the consequences of PPAR*γ* loss of function. Mutations in human PPAR*γ*-coding sequence have been found to cause lipodystrophy (an underdevelopment of adipose tissue). The substantial reductions in adipose tissue mass have been associated with severe insulin resistance and often with hepatosteatosis [8, 32]. An insufficient adipose tissue capacity to buffer dietary FAs, with consequent lipotoxicity due to deposition of TG and acyl-CoA in insulin-sensitive tissues, has been underlined as causative factor for insulin resistance [32]. Moreover, adipose tissue loss has been considered critical for the development of hepatic steatosis in JAK2L mice [20] and in mouse models of severe lipodystrophy [49, 50]. A number of reviewed studies support the correlation between general PPAR*γ*-deficiency and severe lipodystrophy accompanied by insulin resistance and hypotension. Insulin resistance, impaired adipogenesis, elevated levels of plasma free FAs and TGs, and decreased levels of both plasma leptin and adiponectin have been observed also in different mouse models of adipocyte-specific PPAR*γ*-knockout [9].

Targeting drosophila tribbles homologue 3 (Trib3), which* in vitro* prevents PPAR*γ* activation, by antisense oligonucleotide (ASO) has been shown to increase white adipose tissue mass by 70%, improving insulin sensitivity primarily in a PPAR*γ*-dependent manner. Cotreatment with the PPAR*γ* antagonist BADGE blunted the expansion of white adipose tissue and abrogated the insulin-sensitizing effects of Trib3 ASO [51]. Recently, Tsukahara et al. connected the reduced adipogenesis and lipid accumulation in 3T3-L1 cells with the inhibition of PPAR*γ*-mediated reporter gene expression by the endogenous PPAR*γ* antagonist cyclic phosphatidic acid (CPA) that binds to the nuclear receptor with nanomolar affinity and high specificity [52]. Moreover, scoparone which decreased TG accumulation in the mature adipocytes has been reported to suppress the differentiation of 3T3-L1 preadipocytes through down-regulation of adipogenic genes by PPAR*γ* inhibition. It has been shown also to inhibit the rosiglitazone-mediated overexpression of PPAR*γ* target genes to near that observed in cells treated with GW9662 [53].

Based on experimental studies reflecting the importance of PPAR*γ* inhibition for the reduced lipid storage capacity of the adipose tissue, we have developed also a toxicity pathway emphasizing the linkage between this MIE and NAFLD (Figure 3). Among the possible intermediate effects is the decreased expression of adiponectin. The regulation of adiponectin, a hormone exclusively expressed in adipose tissue and recognized by hepatic adiponectin receptors 1 and 2, is under PPAR*γ* control. Besides the improvement of insulin signaling via IRS-1 (insulin receptor substrate 1), adiponectin exerts its effect by enhanced *β*-oxidation of fatty acids through activation of PPAR*α* and phosphorylation of AMPK (5′-adenosine monophosphate-activated protein kinase). The last has been implicated both in reduction of malonyl-CoA-mediated inhibition of *β*-oxidation and in lowering of triglyceride and cholesterol synthesis via suppression of SREBP-1 (Sterol regulatory element-binding protein-1) and ChREBP (Carbohydrate-responsive element-binding protein) [54]. Adiponectin-dependent activation of the AMPK signaling pathway and its role for the lipid metabolism have been confirmed to promote lipid oxidation, suppress lipid synthesis, and reduce hepatic lipid accumulation also in bovine hepatocytes cultured* in vitro* [55]. More importantly, hepatic steatosis has been associated with hypoadiponectinemia. In a study on obese adolescents, hypoadiponectinemia and significantly decreased expression of PPAR*γ*2 in the subcutaneous adipose tissue were associated with high liver fat content, as well as with insulin resistance. An inverse relationship was observed between plasma adiponectin or PPAR*γ*2 expression and hepatic fat content. Adiponectin expression was also positively related to PPAR*γ*2 expression [56]. Shrinkage and reduced secretion of adiponectin by adipose tissue have been shown to initiate a dramatic partitioning of lipid into livers of foz/foz mice [24]. Treatment with 4-hydroxynonenal has been reported to increase adiponectin gene expression, which paralleled elevated PPAR*γ* gene expression and transactivity. As T0070907 (PPAR*γ* antagonist) has been shown to reverse both effects, a critical role of the receptor in this process has been proposed [57]. Recently, it has been reported that eicosapentaenoic acid (EPA) and its metabolite 15d-PGJ_3_ could increase adiponectin secretion in 3T3-L1 adipocytes, partially mediated by PPAR*γ* [58].

In addition to impaired adiponectin secretion, other toxicity pathways in adipocytes have been outlined within the proposed MoA. As already discussed, LD proteins are known to play important regulatory roles in the remodeling (fragmentation, shrinkage, expansion, and/or fusion) of LDs. Reduced expression of LD proteins, transcriptionally regulated by PPAR*γ* (FSP27/CIDEC, Plin1), has been linked to increased adipocyte lipolysis leading to elevated concentration of circulating FAs, insulin resistance, and ectopic lipid deposition in hepatocytes [44, 59].

Gaemers et al. [23] reported the role of the compromised metabolic function of inflamed white adipose tissue in overfeeding mouse models of NAFLD with significant decrease in the expression of PPAR*γ* and its target proteins involved in lipid uptake: CD36 and aP2. Recently, various plant-derived agents (scoparone and extracts from* Zanthoxylum piperitum DC* and* Petalonia binghamiae thalli*) have been shown to inhibit* in vitro* adipocyte differentiation as well as TG accumulation in the mature adipocytes by decreasing the expression of PPAR*γ* [60] and its adipocyte-specific target genes (aP2, CD36/FAT) [53, 61]. Lactic acid bacteria isolated from Korean pickled fish markedly decreased the expression level of PPAR*γ*, aP2, and CD36 and significantly decreased intracellular TG storage [62]. Nuclear factor erythroid 2-related factor 2 has been shown to decrease PPAR*γ* and aP2 expression in mouse embryonic fibroblasts, while in Lep (ob/ob) mice, it inhibited the lipid accumulation in white adipose tissue, suppressed adipogenesis, induced insulin resistance, and increased hepatic steatosis [63].

PPAR*γ* has been recently shown to play a regulatory role in inflammatory and immune responses. Luconi et al. [12] reviewed different mechanisms of action of PPAR*γ* some of which included repression of NFkB pathway. PPAR*γ* has been shown to interfere with transcription of target genes either by direct interaction with NFkB preventing its binding to specific responsive elements on target genes, or by competing for common coactivators. PPAR*γ* maintained inflammation-related genes in a repressed state through blocking the pro-inflammatory stimulus-induced clearance of corepressor complexes on target genes. Ligand-dependent SUMOylation of PPAR*γ* has been reported to induce the expression of IKB*α* (the inhibitory subunit of the NF-*κ*B complex in the cytoplasm) [12, 46]. PPAR*γ*-dependent down-regulation of NF-*κ*B pathway explained the anti-inflammatory action of the PPAR*γ* activator resolvin D1 in lung, partially reversed by GW9662 [64]. Recently, PPAR*γ* activation by bezafibrate has been implicated in the reduction of white adipose tissue inflammatory state [65].

All these data support the prosteatotic role of PPAR*γ* inhibition in adipose tissue, as a possible MIE leading to NAFLD. The decreased adipogenesis and the resulting undesirable changes in adipose lipid-buffering capacity result in changes of plasma free FA and adiponectin levels. These changes were underlined as causative factors for decreased hepatic FA oxidation, increased FA synthesis, and elevated flow of FA into the liver. As already discussed, excessive liver triglyceride accumulation could be a consequence of elevated FA uptake. The latter could also increase the cytosolic free FA pool—an intermediate event preceding the generation of multiple fatty acids—derived mediators of lipotoxicity [54, 66]. The resulting oxidative stress and inflammation comprise a manifestation of the lipotoxic hepatocellular injury associated with NASH [67].

## 4. Concluding Remarks and Perspectives

The concerns about the safety profile of PPAR*γ*-targeting xenobiotics of either synthetic or natural origin are rooted in the risk of developing adverse outcomes upon prolonged treatment. Both agonists and antagonists could reinforce improper and/or ectopic induction/suppression of PPAR*γ*-responsive genes related to lipid metabolism and inflammation with consequent development of NAFLD. The better understanding of the MIE would allow for the definition of the properties of chemicals inducing the perturbation, such as bioavailability, structural requirements (especially for receptor binding), and metabolic transformation [5]. In this regard, an important question to deal with is to identify the primary site of action of toxicants at a tissue level. We propose two probable toxicological MoAs from PPAR*γ* ligand binding to nonalcoholic fatty liver disease: (1) PPAR*γ* activation in hepatocytes and (2) PPAR*γ* inhibition in adipocytes. Increased cellular free FA uptake, exceeding the adaptive pathways of hepatic lipid export and catabolism, could be a prerequisite for adipogenic transformation of hepatocytes. Among the PPAR*γ* target proteins, CD36 is outlined as an essential prosteatotic factor confirmed by most experimental evidence. In adipocytes, PPAR*γ* ligand-dependent inhibition may decrease FA storage capacity of adipose tissue with secondary effects on hepatic FA uptake, synthesis, and *β*-oxidation, facilitating development of NAFLD. The activity of PPAR*γ* apart from its transcriptional regulation, cell-specific expression pattern of its cofactors and their insufficiently understood interactions, depends also on the posttranslational modifications of the receptor, availability of RXR*α* (forming a heterodimer with PPAR*γ*), status of the target genes' promoters, presence of endogenous ligands, regulation of the receptor degradation, and its cellular localization [13, 68–71]. Combination of all these dependencies with the complex cross-talk between different signal transduction pathways makes the evaluation of PPAR*γ*-mediated toxicity pathways within the established MoAs a challenging task. Developing of MoA/AOP as dynamic entities that can be continually updated and refined [5] is the first step towards the building of new generation predictive models of liver toxicity. Development of such models and their application to chemicals lacking extensive* in vivo *testing regarding NAFLD strongly depends on the presence of test results from* in vitro *and/or* in silico* assays as well as of datasets that detail the effects of these chemicals on the whole organisms [72]. Collectively, the reviewed* in vivo *and* in vitro *studies suggest that until a critical evaluation of potential adverse health hazards is performed, extreme caution should be exerted in long-term application of PPAR*γ* modulators. The transcriptional networks and the affected metabolic and signaling pathways in pathological conditions, such as NAFLD, deserve to be further addressed in order to improve risk assessment by the PPAR*γ* targeting strategies. Other major tasks would be the description of pathways from additional transcriptional regulators controlling PPAR*γ* expression or activity as well as gaining insights into the molecular basis and the pathophysiological relevance of different coactivator recruitment following the ligand-binding.

## Supplementary Material

Supplementary Table: Classifies the selected 72 papers according to the studied subjects and experimental approaches.

## Figures and Tables

**Table 1:** Main findings extracted from selected scientific papers supporting the prosteatogenic role of FAT/CD36 in the MoA from PPAR*γ* dysregulation to NAFLD.

Species	PPAR*γ* related strain characteristics	Diet	Experiment type	Gene manipulation	Pharmacological treatment		Endpoints			Reference
					Agent	Type	PPAR*γ*	CD36	NAFLD biomarkers	
Human	NASH patients							+	+	[15]

Mouse	Wild type	HFD					+		+	[16]
	Liver PPAR*γ* deficient line	HFD					0		0	
	Wild type	CD		PPAR*γ* transfected			+	+	+	
	Liver PPAR*γ* deficient line	CD		PPAR*γ* transfected			+	+	+	
**Mouse**		** **	**Hepatocytes**	**PPAR*γ*** ** transfected**	** **	** **	+	+	+	
		** **			**Rosiglitazone**	**Synthetic agonist**	+	++	++	
		** **			**Palmitate**	**Endogenous metabolite**	+	++	++	

Mouse	Functional PPAR*γ*	HFD					+	+	+	[17]
	PPAR*γ* knockout	HFD					0/+	0/+	0/+	
**Mouse**	**Functional PPAR*γ***	** **	**Tissue slices**	** **	**Oleic acid**	**Endogenous agonist**	** **	** **	+	
	**Functional PPAR*γ***	** **		** **	**Rosiglitazone**	**Synthetic agonist**	** **	** **	+	
	**PPAR*γ*** ** knockout**	** **		** **	**Oleic acid**	**Endogenous agonist**	** **	** **	**0**	
	**PPAR*γ*** ** knockout**	** **		** **	**Rosiglitazone**	**Synthetic agonist**	** **	** **	**0**	
	**Functional PPAR*γ***	** **	**Hepatocytes**	** **	**BADGE**	**Synthetic antagonist**	** **	** **	−	
	**Functional PPAR*γ***	** **		** **	**Oleic acid **+** BADGE**	**Endogenous agonist +Synthetic antagonist**	** **	** **	0/+	

Mouse	Insulin-resistant mice	CD					+	+	+	[18]
	Control mice	CD			Pioglitazone	Synthetic agonist			0	
	Insulin-resistant mice	CD			Pioglitazone	Synthetic agonist	0	+	++	

Mouse	Wild type	HFD-safflower oil					0/+	0	0/+	[19]
	Wild type	HFD-butter					+	+	+	
	Wild type	HFD-safflower oil		PPAR*γ*2 knockdown			+	0/+	0/+	
	Wild type	HFD-butter		PPAR*γ*2 knockdown			+	0/+	0/+	
	Wild type	CD		PPAR*γ* transfected			+	+	+	

Mouse	JAK2L-tyrosine kinase deficient	CD					+	++	++	[20]
	Wild type	CD			GW9662	Synthetic antagonist	0	0	0	
	JAK2L-tyrosine kinase deficient	CD			GW9662	Synthetic antagonist	+	+	+	

Mouse	Liver SMS2-overexpressing transgenic line	CD						+	0/+	[21]
	lSMS2-deficient knockout line	CD						−	0/−	
	Wild type	HFD					1	1	+	
	Liver SMS2-overexpressing transgenic line	HFD					+	+	++	
	lSMS2-deficient knockout line	HFD					−	−	−	
	Liver SMS2-overexpressing transgenic line	HFD			GW9662	Synthetic antagonist			−	
**Human**		** **	**Huh7 hepatoma cells**	** **	**Ceramide**	**Endogenous suppressor**	−	−		

Mouse	Wild type	CD		Fbw7 knockdown			+	+	++	[22]
	Wild type	CD		Fbw7/PPAR*γ*2 double knockdown			0/−	0/+	+	
	Wild type	CD		Fbw7 transfected			−	−	0/−	
**Mouse**	**Wild type**	** **	**Hepatocytes **	**Fbw7 knockdown**	** **	** **	+	+	+	

Mouse	Wild type	HFD					+	+	+	[23]
	Wild type	HFD, liquid, overfeeding					++	++	++	

Mouse	Wild type	HFD					0/+	0/+	0/+	[24]
	Obese, hypercholesterolemic, diabetic foz/foz mice	CD					+	+	0/+	
	Obese, hypercholesterolemic, diabetic foz/foz mice	HFD					+	++	+	

Legend: Bold:  *in vitro* experiments; CD: control diet, HFD: high-fat diet; endpoints: empty cells: endpoint not determined, +: increase, −: decrease, 0: no effect, 1: controls taken for 100%; 0/+ and 0/− are used in cases where a clear-cut decision about the reported effects could not be done.
