# Supplementary material for: Modes-of-Action Related to Repeated Dose Toxicity: Tissue-Specific Biological Roles of PPARγ Ligand-Dependent Dysregulation in Nonalcoholic Fatty Liver Disease
Source: PPAR Res. 2014 Mar 18;2014:432647. doi: 10.1155/2014/432647 (PMC3977565; doi:10.1155/2014/432647)
Supplement: Supplementary file 1 — Supplementary Table: Classifies the selected 72 papers according to the studied subjects and experimental approaches. [file 432647.f1.pdf]

SUPPLEMENTARY MATERIAL

Supplementary table 1: Classification of the selected papers according to the experimental subjects and approaches. Reference numbers correspond to those in the reference list of the article.

Reference №	Experimental subject				Experimental approach								AOP-related papers	Background-related papers
	Human patients	Human cell culture	Animal in vivo	Animal cell culture	PPARγ overexpression	PPARγ overexpression and pharmacological treatment	PPARγ knockout / knockdown	PPARγ knockout / knockdown and pharmacological treatment	Pharmacological treatment	Diet manipulation	Gene manipulation of PPARγ upstream proteins	Gene manipulation of PPARγ upstream proteins and pharmacological treatment		
1													✓	
2													✓	
3													✓	
4													✓	
5													✓	
6														✓
7													✓	
8														✓
9														✓
10														✓
11														✓
12														✓
13														✓
14														✓
15	✓													
16			✓	✓	✓	✓				✓				
17			✓	✓			✓	✓		✓				
18			✓						✓					
19			✓		✓		✓			✓				
20			✓						✓					
21		✓	✓						✓	✓				
22			✓	✓			✓				✓			
23			✓							✓				
24			✓							✓				
25														✓
26														✓
27														✓
28	✓													
29			✓	✓			✓	✓						
30														✓
31			✓		✓						✓	✓		
32														✓
33		✓	✓							✓	✓	✓		
34														✓
35			✓		✓		✓			✓	✓			
36		✓									✓			
37			✓							✓				
38														✓
39														✓
40														✓
41														✓
42														✓
43														✓
44														✓
45														✓
46														✓
47														✓
48				✓					✓					
49			✓	✓						✓	✓			
50			✓	✓							✓	✓		
51			✓							✓	✓	✓		
52		✓	✓	✓					✓					
53				✓					✓					
54														✓
55		✓												
56	✓													

57			✓	✓					✓	✓				
58			✓	✓					✓	✓				
59														✓
60			✓	✓					✓	✓				
61			✓	✓					✓	✓				
62				✓					✓					
63			✓	✓					✓	✓	✓			
64			✓						✓					
65			✓						✓	✓				
66														✓
67														✓
68														✓
69														✓
70														✓
71														✓
72													✓	
Total	3	5	25	15	4	1	5	2	14	17	9	4	7	32
